# Transcriptome Highlights Cannabinol Modulation of Mitophagy in a Parkinson’s Disease In Vitro Model

**DOI:** 10.3390/biom13081163

**Published:** 2023-07-25

**Authors:** Agnese Gugliandolo, Santino Blando, Stefano Salamone, Federica Pollastro, Emanuela Mazzon, Simone D’Angiolini

**Affiliations:** 1 IRCCS Centro Neurolesi “Bonino-Pulejo”, Via Provinciale Palermo, Contrada Casazza, 98124 Messina, Italy; agnese.gugliandolo@irccsme.it (A.G.); santino.blando@irccsme.it (S.B.); simone.dangiolini@irccsme.it (S.D.); 2 Department of Pharmaceutical Sciences, University of Eastern Piedmont, Largo Donegani 2, 28100 Novara, Italy; salamone.ste@gmail.com (S.S.); federica.pollastro@uniupo.it (F.P.)

**Keywords:** Parkinson’s disease, mitochondria, mitophagy, cannabinol

## Abstract

Parkinson’s disease (PD) is a neurodegenerative disorder characterized by the loss of dopaminergic neurons in the substantia nigra and the accumulation of α-synuclein aggregates, known as Lewy bodies. It is known that mitochondria dysfunctions, including impaired localization, transport and mitophagy, represent features of PD. Cannabinoids are arising as new therapeutic strategies against neurodegenerative diseases. In this study, we aimed to evaluate the potential protective effects of cannabinol (CBN) pre-treatment in an in vitro PD model, namely retinoic acid-differentiated SH-SY5Y neuroblastoma cells treated with 1-methyl-4-phenylpyridinium (MPP^+^). With this aim, we performed a transcriptomic analysis through next-generation sequencing. We found that CBN counteracted the loss of cell viability caused by MPP^+^ treatment. Then, we focused on biological processes relative to mitochondria functions and found that CBN pre-treatment was able to attenuate the MPP^+^-induced changes in the expression of genes involved in mitochondria transport, localization and protein targeting. Notably, MPP^+^ treatment increased the expression of the genes involved in PINK1/Parkin mitophagy, while CBN pre-treatment reduced their expression. The results suggested that CBN can exert a protection against MPP^+^ induced mitochondria impairment.

## 1. Introduction

Parkinson’s disease (PD) is the second most common neurodegenerative disorder after Alzheimer’s disease. The main features of PD are the loss of dopaminergic neurons in the substantia nigra, resulting in striatal dopamine deficiency, and the abnormal deposition of α-synuclein aggregates in the cytoplasm of neuronal cells, known as Lewy bodies. PD patients mainly show motor symptoms, such as rest tremor, bradykinesia, rigidity and postural instability, but also non-motor symptoms [1].

Mitochondria dysfunction is a well-known pathogenic mechanism involved in PD. PD genetics have indicated an involvement of mitochondria in PD. Indeed, mutations in the genes encoding for PINK1 and Parkin, which play a role in mitochondria functions, were associated with early onset autosomal recessive PD [2]. Moreover, toxins that inhibit the mitochondrial complexes are used to induce PD models [3]. One of these toxins is 1-methyl-4-phenylpyridinium (MPP^+^), a 1-methyl-4-phenyl-1,2,3,6-tetrahydropyridine (MPTP) metabolite. MPP^+^ inhibits mitochondrial complex I, causing, as a consequence, a reduction in ATP production and oxidative stress [3].

Deficits in the mitochondrial respiratory chain, especially in complex I, were reported in post-mortem brains of sporadic PD patients more than 30 years ago [4]. Mitochondrial homeostasis and turnover are important in particular for neuronal cells. Mitochondria are a highly dynamic organelle that are transported in an anterograde manner in neurons, from the cell body to the processes in the cells, where there is a high energy demand, or retrogradely back to the cell body, allowing the removal of damaged mitochondria [5]. Mitophagy, which refers to the process involved in the removal of damaged mitochondria, appears to be altered in PD patients and models. Moreover, PINK1 and Parkin are key players in mitophagy [6].

*Cannabis sativa* L. contains several bioactive compounds that have demonstrated health promoting effects. Specifically, cannabinoids were reported to be promising in the treatment of neurodegenerative diseases, including PD [7]. Δ^9^-tetrahydrocannabinol (Δ^9^-THC) and cannabidiol (CBD) are the most abundant cannabinoids and for this reason also the widely studied. However, minor phytocannabinoids, such as cannabinol (CBN), have also shown important biological and neuroprotective effects [8]. CBN treatment significantly delays disease onset in an amyotrophic lateral sclerosis model, but with no effects on survival [9], and it showed antioxidant effects [10]. Interestingly, cannabinoids can modulate several mitochondrial processes, including mitochondrial dynamics and mitophagy, bioenergetic metabolism and apoptosis [11]. A recent report indicated that CBN targets mitochondria and preserves their functions such as redox regulation, membrane potential, bioenergetics, biogenesis, and modulation of fusion/fission dynamics [12].

In this study, we aimed to evaluate the potential protective effects of CBN in an in vitro model of PD, focusing on mitochondria functions, given the major role of mitochondria in PD pathogenesis. With this aim, we pre-treated retinoic acid (RA)-differentiated SH-SY5Y neuroblastoma cells with CBN, exposed them to MPP^+^, and performed a transcriptomic analysis using next-generation sequencing (NGS).

## 2. Materials and Methods

### 2.1. Plant Material and Synthesis of CBN

Non-psychoactive *Cannabis sativa* L. plant material, belonging to the III chemotype, was purchased from Canvasalus Srl (Monselice, Italy). A voucher specimen (Cs-CBD/03/2021) of the vegetal material is stored in Novara laboratories.

**Figure 1:**
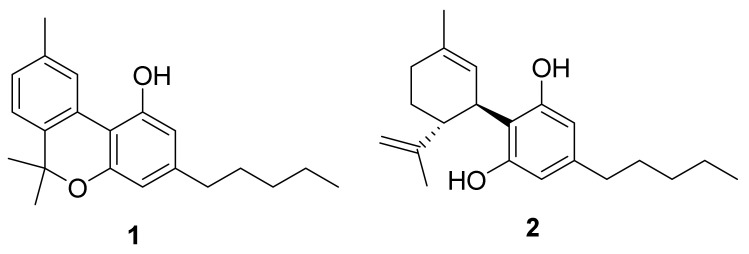
Structure of CBN (**1**) and CBD (**2**).

CBN (**1**) was synthetized starting from 100 mg of CBD (**2**) (Figure 1, created with the software ChemDraw 19), obtained by extraction and purification from non-woody *C. sativa* aerial parts, following the protocol described by [13].

The reaction led to obtaining 80 mg of residue that was further purified by HPLC (silica petroleum ether—EtOAc gradient from 95:5 to 85:15) affording 67 mg of pure CBN (**1**) identified by ^1^H NMR data (Figure S1), according to literature [14]. ^1^H 400 MHz NM spectra were measured on Bruker 400 spectrometers (Bruker^®^, Billerica, MA, USA). Chemical shifts were referenced to the residual solvent signal (CDCl_3_: δH = 7.26). Silica gel 60 (70–230 mesh), used for low-pressure chromatography, was purchased from Macherey-Nagel (Düren, Germany). Purifications were monitored by TLC on Merck 60 F254 (0.25 mm) plates, visualized by staining with 5% H_2_SO_4_ in EtOH and heating. Chemical reagents and solvents were from Aldrich (Darmstadt, Germany) and were used without any further purification unless stated otherwise. HPLC JASCO Hichrom, 250 mm × 25 mm, silica UV–vis detector-2075 plus (Oklahoma, Japan).

### 2.2. Cell Culture and Treatment

The SH-SY5Y cell line was obtained from the American Type Culture Collection (ATCC) (Manassas, VA, USA). Cells were cultured using Dulbecco’s Modified Eagle’s Medium/Nutrient Mixture F-12 Ham (DMEM/F12) medium (Sigma-Aldrich, Saint Louis, MO, USA), containing 10% Fetal Bovine Serum (FBS) (Sigma-Aldrich, Saint Louis, MO, USA), 1% glutamine, and 1% penicillin-streptomycin. In order to induce neuronal differentiation, SH-SY5Y cells were treated with 10 µM RA for 5 days. At the end of the 5-day-differentiation, the medium was replaced with fresh medium with or without CBN at the concentration 10 or 20 µM (dissolved in DMSO; final DMSO concentration <0.1%). A previous report showed no cytotoxicity at these concentrations [12]. After 24 h of pre-treatment, the medium was changed with fresh medium with 1 mM MPP^+^ dissolved in water for 48 h. The MPP^+^ concentration and time of exposure were chosen on the basis of a previous report [15]. Control cells were incubated with DMEM/F12 medium supplemented with 10% FBS.

### 2.3. MTT

In order to evaluate cell viability, SH-SY5Y cells were cultured in 96-well plates, treated as reported in the previous paragraph and MTT assay was carried out. At the end of the treatment, cells underwent medium replacement with Thiazol Blue Tetrazolinium Bromide (MTT) at a concentration of 0.5 mg/mL (Sigma-Aldrich Merck KGaA, Darmstadt, Germany) for 4 h at 37 °C. The formed crystals were dissolved using acidic isopropanol. The optic density was measured spectrophotometrically using microplate reader Victor NIVO^TM^ (PerkinElmer, Waltham, MA, USA). Experiments were carried out in triplicate.

### 2.4. Transcriptomic Analyses and Bioinformatic Processing

At the end of the treatment, control cells, cells treated with MPP^+^ and cells pre-treated with CBN 10 µM and after with MPP^+^ were harvested and Maxwell^®^ RSC simplyRNA Cells Kit (Promega, Madison, WI, USA) was used to extract RNA, following the manufacturer’s instruction. TruSeq RNA Exome protocol (Illumina, San Diego, CA, USA) was used for the library preparation. The library was analyzed using the Illumina instrument NextSeq 550 Dx.

To evaluate the quality of readings analyzed in this work, we used FastQC v0.11.9 (Babraham Institute, Cambridge, UK) [16]. Using Trimmomatic v.0.40-rc1 (Usadel Lab, Aachen, Germany) [17], it was possible to remove the adapters and the readings with a low-quality score. After the trimming phase, the remaining reads were aligned to a reference genome using STAR RNA-seq aligner 2.7.10a_alpha_220207 (New York, NY, USA) [18]. The genome used as reference for the alignment is hg38 v39 from GENCODE. After completing the alignment phase, it was possible to obtain transcript counts for each gene using HTSeq v. 0.13.5 [19]. The list of transcript counts was used for differential gene expression analysis with DESeq2 library v. 1.36.0 [20] in R v. 4.2.0 (R Core Team). To correct the *p*-value obtained from DESeq2 analysis, we used the Benjamini–Hochberg correction, setting the threshold of the q-value to 0.05. With the differentially expressed genes (DEGs) list, it was possible to perform the gene ontology (GO) overrepresentation analysis using the R package clusterProfiler v. 4.4.3 [21]. Inspection of supplementary specific biological process was implemented through the AmiGo2 database [22]. Additional information about the altered pathway in which DEGs were involved was obtained using the KEGG database [23].

### 2.5. Protein Extraction and Western Blot

At the end of the treatments, proteins were extracted using the NE-PER™ Nuclear and Cytoplasmic Extraction Reagents (Thermo Scientific™, Waltham, MA, USA), according to the manufacturer’s instructions. Protein concentrations were measured with the Bradford assay (Bio-Rad, Hercules, CA, USA). Twenty-five micrograms of proteins were heated for 5 min at 95 °C, resolved by SDS-polyacrylamide gel electrophoresis (SDS-PAGE) and transferred onto a PVDF membrane (Immobilon–P, Millipore, Burlington, MA, USA). Membranes were blocked in 5% skim milk in TBS for 1 h at room temperature followed by incubation overnight at 4 °C with the following antibodies: anti-LC3 antibody (cat: #12741; 1:500; Cell Signaling Technology, Danvers, MA, USA); anti-PINK1 (cat: P0076; 1:1000; Sigma-Aldrich, Saint Louis, MO, USA); anti-p62 (cat: #39749; 1:1000; Cell Signaling Technology, Danvers, MA, USA); anti-β-actin (cat: sc-47778; 1:1000; Santa Cruz Biotechnology, Dallas, TX, USA). Then, membranes were washed in TBS 1 × and incubated with HRP-conjugated anti-rabbit (cat: sc2357; 1:2000; Santa Cruz Biotechnology Inc., Dallas, TX, USA) or anti-mouse IgG secondary antibodies (cat: SA1-72021; 1:2000; Invitrogen, Waltham, MA, USA) for 1 h at room temperature. The relative expression of protein bands was visualized using an enhanced chemiluminescence system (Luminata Western HRP Substrates, Millipore, Burlington, MA, USA) and protein bands were acquired and quantified with ChemiDoc™ XRS+ System (Bio-Rad, Hercules, CA, USA) and a computer program (ImageJ 1.53t software), respectively. The expression of LC3II was normalized on the expression of LC3I. However, we incubated the blot with GAPDH HRP-conjugated antibody (cat: #3683; 1:1000; Cell Signaling Technology, Danvers, MA, USA) as a loading control. The uncropped blots for LC3 and GAPDH loading control are available in supplementary Figure S2. The uncropped blot for PINK1 and p62 are shown in the supplementary Figures S3 and S4, respectively.

Blots are representative of three independent experiments.

### 2.6. Statistical Analysis

The MTT and Western blot statistical analysis was performed using GraphPad Prism 9.0 software (GraphPad Software, La Jolla, CA, USA). The multiple comparison was performed using a one-way ANOVA test followed by Bonferroni post hoc test. A *p*-value less than or equal to 0.05 was considered statistically significant. The results are expressed by mean ± standard deviation (SD).

## 3. Results

### 3.1. CBN Attenuated the Loss of Cell Viability Induced by MPP^+^ Treatment

**Figure 2:**
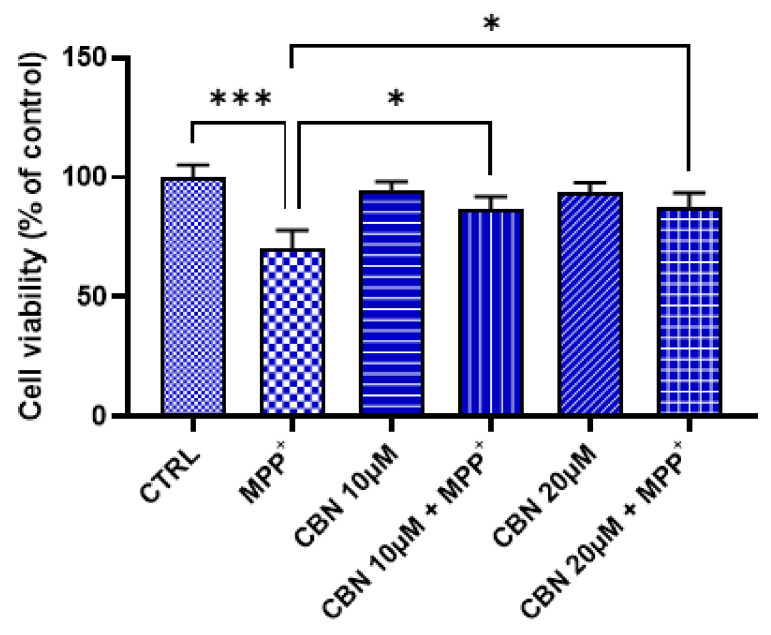
CBN was able to attenuate the MPP^+^-induced loss of cell viability. CBN was not cytotoxic at both concentrations. An amount of 1 mM MPP^+^ reduced cell viability, but CBN pre-treatment counteracted the MPP^+^ toxicity (*n* = 3). Of note, MTT assay evaluates the number of metabolically active cells, which can be used to estimate the cell viability. Possible variables and confounding factors should be taken into consideration when interpreting the results. * *p* < 0.05; *** *p* < 0.001.

At first, we evaluated if CBN was cytotoxic and if it can counteract the MPP^+^-induced loss of cell viability. Evaluation of the cell viability evidenced that CBN, at both 10 and 20 µM concentrations, was not cytotoxic. On the contrary, the treatment with 1 mM MPP^+^ for 48 h significantly induced a loss of cell viability. However, CBN at both concentrations was able to attenuate the loss of cell viability induced by MPP^+^ treatment (Figure 2). Given the similar effects on cell viability, we decided to perform the other experiments using the dose 10 µM of CBN.

It is important to notice that MTT assay is a colorimetric assay performed to measure the metabolic activity in living cells, but it is widely used as a measurement of cell viability. The test is based on the reduction of a water soluble, yellow tetrazolium salt MTT to a non-water-soluble purple formazan crystals by metabolically active cells, and specifically by NAD(P)H-dependent cellular oxidoreductase enzymes. The formed formazan crystals are then dissolved resulting in a colored solution, whose optical density is measured. The intensity of the color is proportional to the number of metabolically active cells, which can be used to estimate the cell viability [24]. However, possible variables and confounding factors should be taken into consideration when interpreting the results [24,25].

### 3.2. CBN Pre-Treatment Modulated DEGs Involved in Mitochondrial Localization and Mitophagy after MPP^+^ Treatment

A transcriptomic analysis was performed to evaluate the transcriptional patterns induced by the pre-treatment with CBN of RA-differentiated SH-SY5Y cells treated with MPP^+^. The first comparison was performed on the control group against the MPP^+^ group (CTRL vs. MPP^+^). This comparison resulted in 15462 DEGs among which, after the treatment with MPP^+^, 8096 were downregulated, while 7366 were upregulated. The second comparison was conducted between the MPP^+^ group against CBN 10 μM + MPP^+^ (MPP^+^ vs. CBN 10μM + MPP^+^). MPP^+^ vs. CBN 10 μM + MPP^+^ resulted in 8301 DEGs, among which 4161 were downregulated in the CBN pre-treated group, while 4140 were found to be upregulated. The biological processes with a significative number of DEGs involved were identified for each comparison through the overrepresentation analysis. Given the involvement of mitochondria in PD, we decided to focus our attention on biological processes related to mitochondrion. Using the advanced research function of the AmiGo2 database, we obtained the list of 192 biological processes related to mitochondrion. Then, we intersected this list with the list of the 469 biological processes resulted overrepresentated in both comparisons. In this way, we obtained a list of the 17 biological processes related to mitochondria functions overrepresented in both our comparisons. The list of the 17 biological processes is reported in Table 1. In order to evaluate which biological process CBN was more efficacious in reversing the alteration of gene expression induced by MPP^+^, we focused our attention on the DEGs that in MPP^+^ vs. CBN 10 μM + MPP^+^ assumed an opposite expression trend compared to the one observed in CTRL vs. MPP^+^ and ordered biological processes according to their ratio. The DEGs for each biological process are available in the Supplementary file Excel S1.

**Figure 3:**
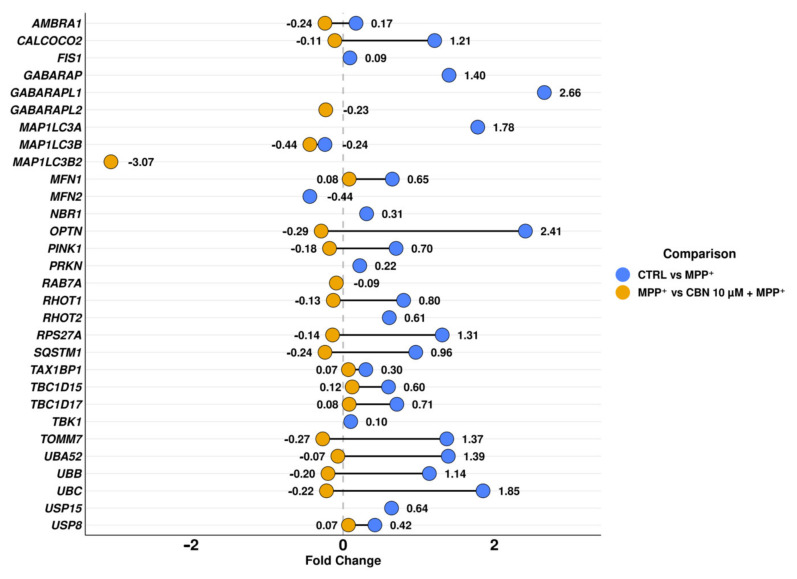
Fold change related to the DEGs involved in PINK1/Parkin mitophagy. For the DEGs common to both comparisons, the distance of the fold change is highlighted.

The biological processes with a higher ratio were mainly related to transport and localization of mitochondria and autophagy and disassembly of mitochondria, a mechanism also known as mitophagy. Given the reported role of mitophagy in PD, we focused our attention on the mitophagy pathway in KEGG, which highlights the cascade of events and the interaction of each protein with the others. On the contrary, biological processes do not give information about protein interactions with the other proteins or the consequentiality of these interactions. In particular, we focused on the PINK1/Parkin mitophagy pathway. The DEGs involved in this pathway with the related fold change for both comparisons are reported in Table 2. In addition, Figure 3 shows the above-mentioned DEGs, to underline the distance between the fold change in the different comparisons, and the graphical position of the DEGs in only one comparison.

### 3.3. Western Blot

**Figure 4:**
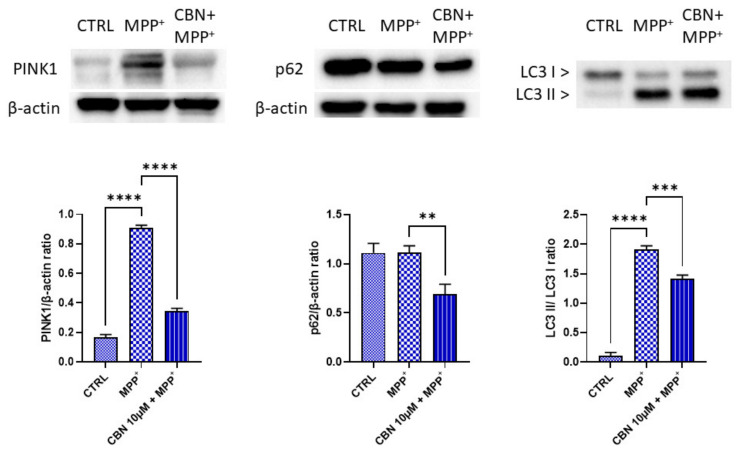
CBN reduced the MPP^+^-induced increase in PINK1, p62 and LC3 II levels. MPP^+^ treatment increased PINK1 and LC3 II levels. CBN pre-treatment was able to reduce PINK1, p62 and LC3 II levels (*n* = 3). ** *p* < 0.01; *** *p* < 0.001; **** *p* < 0.0001.

Western blot analysis was performed to evaluate the expression of PINK1, p62 and LC3. MPP^+^ treatment increased both PINK1 and LC3 II protein levels. Interestingly, the pre-treatment with CBN significantly reduced PINK1, p62 and LC3 II levels (Figure 4).

## 4. Discussion

Neurons represent high energy demanding cells, consuming about 20% of resting energy of the body provided by mitochondria [26,27]. For this reason, mitochondria represent an important organelle for neuronal physiology and their homeostasis, proper functioning, dynamics and distribution are essential [26]. Mitochondrial dysfunctions are a known feature of PD [28].

In this study, we evaluated, using NGS analysis, the protective effects of CBN in an in vitro PD model, namely RA-differentiated neuroblastoma SH-SY5Y cells treated with MPP^+^. This cell line is widely used to induce PD models [3]. In particular, differentiated SH-SY5Y cells represent a better PD model compared to undifferentiated cells. Differentiation induces neurite outgrowth, a morphological similarity to neurons in the human brain and the expression of neuronal markers [29]. Various differentiating agents were used to induce SH-SY5Y differentiation, but RA was the most commonly used in studies involving PD models [30]. A report investigated the molecular phenotype of RA differentiated SH-SY5Y cells using genome wide transcriptional profiling demonstrating that RA induced a neuronal differentiation program in SH-SY5Y cells toward a predominantly mature DAergic-like neurotransmitter phenotype [31], indicating its suitability to induce PD in vitro models. Moreover, given that it represents the most used PD model, the use of this model makes the comparison with previously published reports easier. Some studies also used RA in combination with other agents, such as BDNF. Indeed, the addition of BDNF promotes molecular polarization in differentiating neuroblastoma cells leading to definitive neurons, which show an enrichment of MAP2 in the dendrite and tau in axons [32,33]. The addition of BDNF to RA led to a homogeneous neuronal population with expression of neuronal markers. However, the phenotype deriving from RA/BDNF differentiation protocol is still controversial [30].

We found that CBN was able to attenuate the MPP^+^-induced loss of cell viability.

GO analysis evidenced that the most important biological processes, in which CBN showed a major opposite modulation in gene expression compared to MPP^+^ treatment, were mainly relative to mitochondria transport, localization and mitophagy.

Mitochondria in neurons are characterized by frequent changes in direction and a non-uniform distribution in soma, axon and dendrites. Defects in mitochondrial transport and localization are implicated in the pathogenesis of several neurological disorders. The mitochondrial mobility depends on a coordination of anterograde and retrograde motor proteins [5], such as Miro, a mitochondrial outer membrane protein that plays a main role in mitochondrial axonal transport and dynamics [34]. In our model, Miro isoforms 1 and 2, encoded by the genes *RHOT1* and *RHOT2*, were upregulated in RA-differentiated SH-SY5Y treated with MPP^+^. A study demonstrated that PD patient fibroblast cell lines fail to remove the isoform Miro1 after depolarization. Interestingly, Miro1 reducer rescued the locomotor deficits and dopaminergic neurodegeneration in PD models. Therefore, Miro1 can be considered a marker and a target for personalized medicine [35]. Interestingly, in our experimental condition, CBN pre-treatment reduced *RHOT1* expression.

Miro can also interact with myosin XIX, which is involved in actin-mediated mitochondrial transport [36]. *MYO19* was downregulated by MPP^+^ and upregulated by CBN. In neuronal cells, MYO19 caused a reduction in mitochondrial run length because it caused a switch from microtubules to actin filaments [37].

Microtubules are dynamic structures stabilized by microtubule-associated proteins (MAPs), such as MAP1B, which was associated with the regulation of retrograde axonal transport of mitochondria [38] and it is a component of Lewy bodies [39]. *MAP1B* was upregulated by MPP^+^ treatment, but downregulated by CBN pre-treatment. We also found a dysregulation of *MARK1,* which is a regulator of microtubule-dependent transport in axons [40]. *MARK1* was downregulated by MPP^+^, but CBN increased its expression.

Fez1 is important for the anterograde axonal mitochondrial transport [41]. NEFL can modulate mitochondrial morphology, fusion and transport, promoting mitochondrial fusion, increase mitochondrial length, and slow mitochondrial mobility [42]. Both *FEZ1* and *NEFL* increased after MPP^+^ treatment, but were downregulated by CBN treatment.

MGARP is located in the inner/cristae membranes and mediates mitochondrial motility. Its overexpression reduced the movement of mitochondria, induced alterations in the structural integrity, cristae shaping and subcellular distribution, also suggesting it plays a role in mitophagy [43]. *MGARP* was upregulated by MPP^+^, while it was downregulated by CBN pre-treatment.

These results can suggest that MPP^+^ treatment induced alteration in mitochondria motility, as suggested by the increase in *RHOT1,* with a possible reduction in movements, suggested by *MGARP* increase. Interestingly, the alterations in mitochondria motility impact both anterograde and retrograde and microtubules and actin mediated movements. CBN counteracted these changes and, in particular, its effect on reducing *RHOT1* expression may be important, given that Miro1 reducer proved to be able to rescue PD neurodegeneration [35].

Other biological processes modulated by CBN regard autophagy of mitochondria, known as mitophagy. MPP^+^ was reported to increase mitophagy [44]. In the basal condition, PINK1 is imported by the TIM and TOM complex in the inner mitochondrial membrane where it is cleaved by presenilin-associated rhomboid-like protease and then degraded [45]. In case of mitochondria depolarization, such as after MPP^+^ treatment, PINK1 is stabilized at the mitochondrial outer membrane (MOM). After its accumulation at MOM, it phosphorylates ubiquitins conjugated with MOM proteins at Ser65. This accumulation recruits Parkin, which shows a high affinity for phosphorylated ubiquitin, inducing its translocation from cytosol to mitochondria. Parkin is an E3 ligases that, when phosphorylated by PINK1, begins to ubiquitinate other MOM proteins [45]. In our experimental model, we found the upregulation of *TOMM7*, shown to be essential for stabilizing PINK1 on the MOM [46]. The genes *PINK1* and *PRKN,* encoding for Parkin, were upregulated by MPP^+^. However, CBN pre-treatment reduced *TOMM7* and *PINK1* expression. The increase in PINK1 level after MPP^+^ treatment and its reduction with CBN were also confirmed with Western blot analysis. In addition, the ubiquitin (UB) *RPS27A*, *UBA52*, *UBB* and *UBC* were upregulated in our model, while they were downregulated by CBN pre-treatment.

It is important to notice that Miro also has a role in regulating mitochondria turnover in neurons, because it arrests damaged mitochondria transport through the PINK1/Parkin pathway, which in turn regulates Miro turnover [34]. Other than Miro, mitofusin (Mfn) 1 and 2 are also ubiquitinated by Parkin, in order to avoid the fusion of damaged mitochondria. We found that MPP^+^ downregulated *MFN2*, while the expression of *MFN1* increased. CBN pre-treatment upregulated *MFN1*. However, Mfn also mediates other processes, such as mitochondria motility, morphology and mitochondria-endoplasmic reticulum cross-talk [47].

The Parkin ubiquitination can be affected by deubiquitinating enzymes (DUBs). In our experimental condition, *USP8* was upregulated by MPP^+^ treatment, associated with a promotion of mitophagy by directly detaching UB from Parkin [48]. It is also upregulated by CBN, but it can be explained by the multiple roles played by USP8, which is an essential DUB involved in cell cycle progression, apoptosis, genomic integrity and regulation of transmembrane receptors [49].

Ubiquitinated MOM proteins can induce the recruitment of autophagy receptors to mitochondria. The autophagy receptors that were linked to mitophagy are p62 encoded by the gene *SQSTM1*, Tax1BP1 encoded by *TAX1BP1*, NDP52 encoded by *CALCOCO2*, optineurin encoded by *OPTN* and NBR1 encoded by *NBR1* [45], which recruit LC3 to start autophagosome formation. All the genes that encode these proteins were upregulated in MPP^+^ treated cells, together with TBK1, which phosphorylates p62, NDP52 and OPTN to increase their affinity for the ubiquitinated proteins [45,50,51]. *SQSTM1*, *OPTN* and *CALCOCO2* were downregulated with CBN. The reduction in p62 by CBN pre-treatment was confirmed by Western blot analysis. It is possible to speculate that in the MPP^+^ group, p62 did not increase significantly because there is a balance between new synthesis and its degradation in the mitophagy pathway. *NBR1* showed no change with the CBN, while *TAX1BP1* was upregulated. The increase in *TAX1BP1*, also in the presence of CBN, may be explained because it was reported to be more specifically expressed in the brain compared to other autophagy receptor proteins, where it is required for the clearance of aggregates [52]. The increased expression of these genes is in line with data showing that NBR1 and p62 were shown to be localized in Lewy bodies [53,54]. OPTN was shown to be enriched in dopamine neurons in the midbrain and its expression increased after rotenone treatment in vivo [55].

All these receptors bind polyubiquitin chains located on mitochondria on one side and LC3 located on the phagosome membranes on the other side. The cytosolic form of LC3, indicated as LC3-I, is conjugated to phosphatidylethanolamine to form LC3-phosphatidylethanolamine conjugate, known as LC3-II, which is recruited to autophagosomal membranes [56]. Different genes encode LC3. In this study *GABARAP, GABARAPL1* and *MAP1LC3A* were found upregulated with the MPP^+^, while *MAP1LC3B* showed a downregulation. CBN pre-treatment decreased *MAP1LC3B*, *MAP1LC3B2* and *GABARAPL2* expression. Western blot analysis confirmed the increased expression of the LC3-II form after treatment with MPP^+^, while CBN reduced its levels.

In this study, we also found that MPP^+^ treatment increased *AMBRA1* expression. It was shown that AMBRA1 can bind Parkin in adult mouse brains and that their interaction strongly increased during prolonged mitochondrial depolarization [57]. Moreover, AMBRA1 can mediate mitophagy binding LC3 [58] and promote PINK1 stability [59]. Notably, CBN pre-treatment decreased *AMBRA1* expression.

We also found the upregulation of *TBC1D15, TBC1D17* and *FIS1* in MPP^+^ treated cells. CBN upregulated *TBC1D15* and *TBC1D17* and downregulated *RAB7A.* TBC1D15 and TBCD17 have a role in autophagosome biogenesis and morphology downstream of Parkin activation. TBC1D15 inhibits Rab7 activity and associates with the mitochondria, binding Fis1, and to the isolation membrane, binding LC3/GABARAP family members. TBC1D17 participates in mitophagy forming homodimers and heterodimers with TBC1D15 [60].

**Figure 5:**
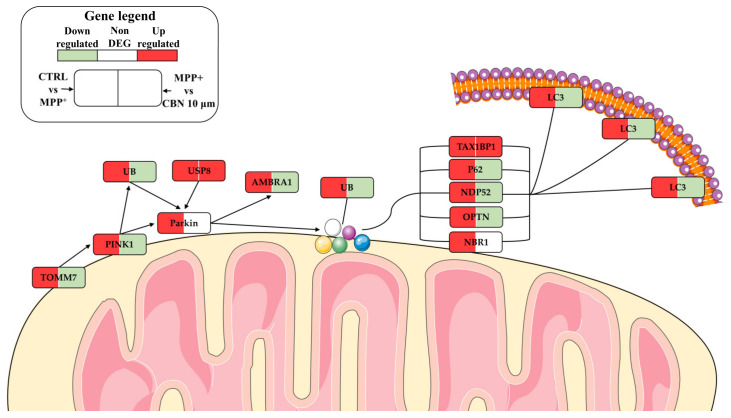
Schematic representation of PINK1/Parkin mitophagy pathway. MPP^+^ increased the expression of the genes involved in mitophagy. On the contrary, CBN pre-treatment decreased their expression. The figure was created using the vector image bank of Servier Medical Art by Servier (http://smart.servier.com/ (accessed on 1 March 2023)). Licensed under a Creative Commons Attribution 3.0 Unported License (https://creativecommons.org/licenses/by/3.0/ (accessed on 1 March 2023)).

A schematic representation of the mitophagy pathway with our DEGs is available in Figure 5.

Among the other modulated biological processes, we also found respiratory chain complex IV assembly. Electron transport chain dysfunctions represent a common feature in PD, also involving complex IV. Electron transport chain dysfunctions not only reduce ATP production but can also influence mitophagy in PD [61].

The other biological processes that were modulated were related to mitochondria organization and protein targeting to mitochondria. Importing protein into mitochondria is a highly regulated process because it is fundamental to prevent the import of abnormal proteins, to remove aberrant proteins and to control the access of specific proteins to respond to cellular demands. Indeed, importing a defective protein can also lead to mitophagy [62]. Notably, CBN was shown to be able to attenuate the MPP^+^-altered expression of the genes involved in protein localization into mitochondria.

Therefore, our results are in line with the known effects of CBN on mitochondria dynamics [12] and deepen the knowledge on the protective effects of CBN on mitochondria homeostasis, showing that CBN is able to attenuate alterations in mitochondria localization, transport and elimination in an in vitro model of PD. To our knowledge, this is the first study demonstrating a protective role of CBN in a PD model, thanks to its capacity to modulate these mitochondrial processes.

The strength of this work is that thanks to transcriptomic analysis, we were able to investigate genes involved in GO and pathways relative to mitochondria functions. Indeed, transcriptomic analysis has the advantages of allowing the analysis of multiple genes altogether in order to have a wider and deeper evaluation of a complex process compared to other techniques. However, a limit of this study is that the present results were obtained in an in vitro PD model and, therefore, should be confirmed in a future in vivo study to further deepen the knowledge on the therapeutic effects induced by CBN, which may represent a new strategy against mitochondrial damage.

## 5. Conclusions

Mitochondria functions need to be strictly regulated to maintain neuron homeostasis and proper physiology. MPP^+^ treatment altered biological processes related mainly to mitochondria localization, transport, protein regulation and mitophagy. Notably, we found that CBN pre-treatment was able to attenuate the pattern of gene expression induced by MPP^+^ in regard to the mentioned processes, suggesting that CBN pre-treatment may exert a protection for mitochondria against MPP^+^ damage.

## Figures and Tables

**Table 1:** Enriched biological processes involved in mitochondrion activity.

GO	Biological Process	Opposite	Total	Ratio
GO:0051654	Establishment of mitochondrion localization	13	29	0.45
GO:0034643	Establishment of mitochondrion localization, microtubule-mediated	11	26	0.42
GO:0047497	Mitochondrion transport along microtubule	11	26	0.42
GO:0008535	Respiratory chain complex IV assembly	10	26	0.38
GO:0000422	Autophagy of mitochondrion	33	88	0.38
GO:0061726	Mitochondrion disassembly	33	88	0.38
GO:1903955	Positive regulation of protein targeting to mitochondrion	12	32	0.38
GO:1903214	Regulation of protein targeting to mitochondrion	16	44	0.36
GO:1903749	Positive regulation of establishment of protein localization to mitochondrion	13	36	0.36
GO:0010823	Negative regulation of mitochondrion organization	18	51	0.35
GO:1903747	Regulation of establishment of protein localization to mitochondrion	17	50	0.34
GO:0010821	Regulation of mitochondrion organization	48	148	0.32
GO:0070585	Protein localization to mitochondrion	40	125	0.32
GO:0072655	Establishment of protein localization to mitochondrion	38	120	0.32
GO:0006626	Protein targeting to mitochondrion	30	101	0.30
GO:0007006	Mitochondrial membrane organization	32	117	0.27
GO:0006839	Mitochondrial transport	47	182	0.26

The 17 biological processes enriched in both comparisons and related to mitochondrion. For each biological process, the number of DEGs that assume an opposite expression trend in the two comparisons, the total number of genes involved in the biological process and the ratio between these two elements is specified. The biological processes shown are reordered based on their ratio.

**Table 2:** DEGs involved in PINK/Parkin mitophagy pathway.

Gene	Fold Change CTRL vs. MPP^+^	*q*-Value CTRL vs. MPP^+^	Fold Change MPP^+^ vs. CBN 10 μM + MPP^+^	*q*-Value MPP^+^ vs. CBN 10 μM + MPP^+^
*AMBRA1*	0.17	3.77 × 10^−11^	−0.24	1.19 × 10^−11^
*CALCOCO2*	1.21	0	−0.11	1.11 × 10^−3^
*FIS1*	0.09	1.08 × 10^−3^	-	-
*GABARAP*	1.40	1.57 × 10^−40^	-	-
*GABARAPL1*	2.66	0	-	-
*GABARAPL2*	-	-	−0.23	1.81 × 10^−6^
*MAP1LC3A*	1.78	1.64 × 10^−199^	-	-
*MAP1LC3B*	−0.24	5.69 × 10^−10^	−0.44	1.45 × 10^−14^
*MAP1LC3B2*	-	-	−3.07	1.03 × 10^−2^
*MFN1*	0.65	3.93 × 10^−182^	0.08	6.60 × 10^−3^
*MFN2*	−0.44	2.30 × 10^−142^	-	-
*NBR1*	0.31	4.19 × 10^−43^	-	-
*OPTN*	2.41	0	−0.29	1.71 × 10^−45^
*PINK1*	0.70	2.08 × 10^−65^	−0.18	1.07 × 10^−3^
*PRKN*	0.22	4.01 × 10^−3^	-	-
*RAB7A*	-	-	−0.09	2.95 × 10^−5^
*RHOT1*	0.80	7.11 × 10^−304^	−0.13	3.69 × 10^−6^
*RHOT2*	0.61	9.55 × 10^−174^	-	-
*RPS27A*	1.31	0	−0.14	2.98 × 10^−133^
*SQSTM1*	0.96	0	−0.24	1.09 × 10^−17^
*TAX1BP1*	0.30	5.27 × 10^−90^	0.07	4.80 × 10^−4^
*TBC1D15*	0.60	2.71 × 10^−148^	0.12	2.95 × 10^−5^
*TBC1D17*	0.71	8.08 × 10^−112^	0.08	3.98 × 10^−2^
*TBK1*	0.10	2.15 × 10^−2^	-	-
*TOMM7*	1.37	0	−0.27	5.58 × 10^−32^
*UBA52*	1.39	0	−0.07	6.76 × 10^−28^
*UBB*	1.14	0	−0.20	0
*UBC*	1.85	0	−0.22	0
*USP15*	0.64	9.36 × 10^−109^	-	-
*USP8*	0.42	2.66 × 10^−74^	0.07	1.66 × 10^−2^

The 30 DEGs involved in PINK1/Parkin mitophagy with relative-fold change and q-value in CTRL vs. MPP^+^ and MPP^+^ vs. CBN 10 μM + MPP^+^. The column Fold Change shows for each DEG the difference in the level of expression computed by log_2_(MPP^+^/CTRL) or log_2_(CBN 10 μM + MPP^+^/MPP^+^). The *q*-Value column was obtained correcting the *p*-value through Benjamini–Hochberg correction. All values were rounded to the second decimal digit.

## Data Availability

The data presented in this study are openly available in the NCBI Sequence Read Archive at BioProject accession number PRJNA945813.

## Supplementary Materials

The following supporting information can be downloaded at: https://www.mdpi.com/article/10.3390/biom13081163/s1, Figure S1: ^1^H NMR of CBN (**1**) in CDCl_3_, 400 MHz; Figure S2: Uncropped blot for LC3 and GAPDH; Figure S3: Uncropped blot for PINK1; Figure S4: Uncropped blot for p62; Excel S1: Inspected DEGs for biological processes.
