# Supplementary material for: Transcriptome Highlights Cannabinol Modulation of Mitophagy in a Parkinson’s Disease In Vitro Model
Source: Biomolecules. 2023 Jul 25;13(8):1163. doi: 10.3390/biom13081163 (PMC10452113; doi:10.3390/biom13081163)
Supplement: Supplementary file 1 [file biomolecules-13-01163-s001.zip › biomolecules-2486961-suppl-Figures.pdf]

Supplementary Files-Figures

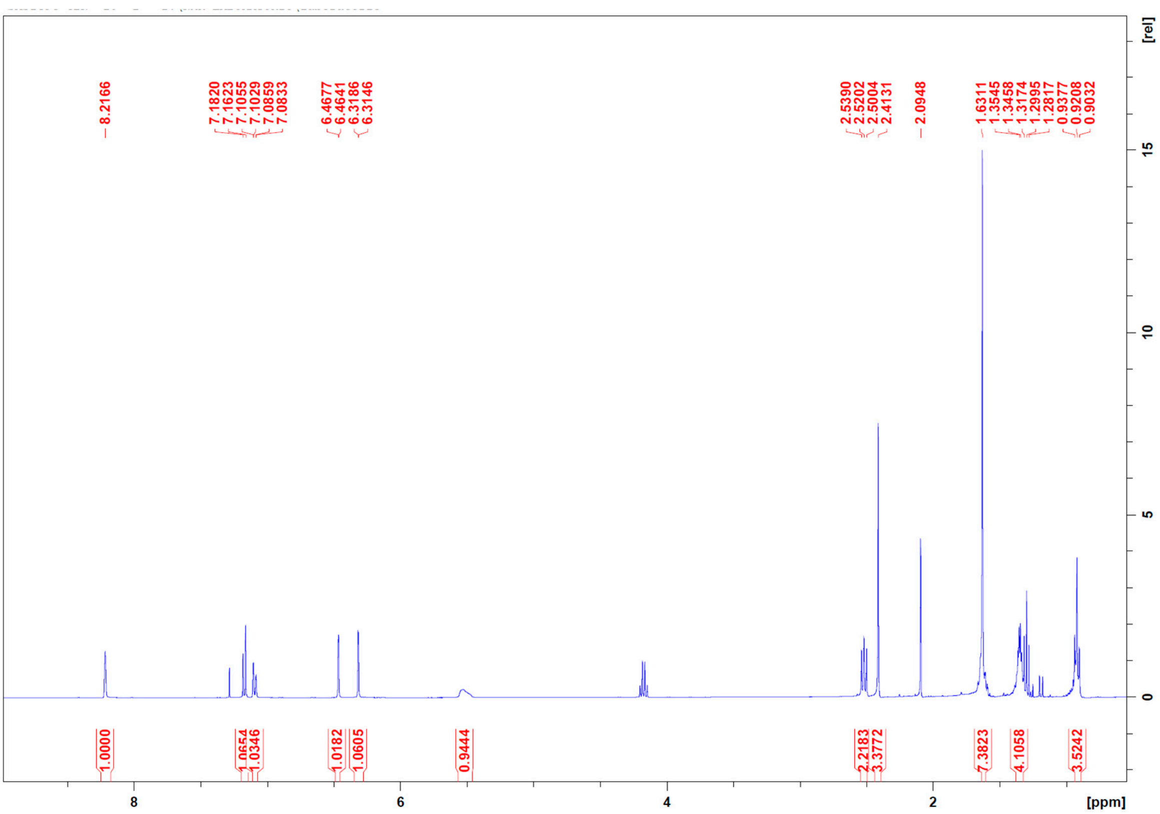

Figure S1: ^{1}H NMR of CBN (1) in $CDCl_3$, 400 MHz.

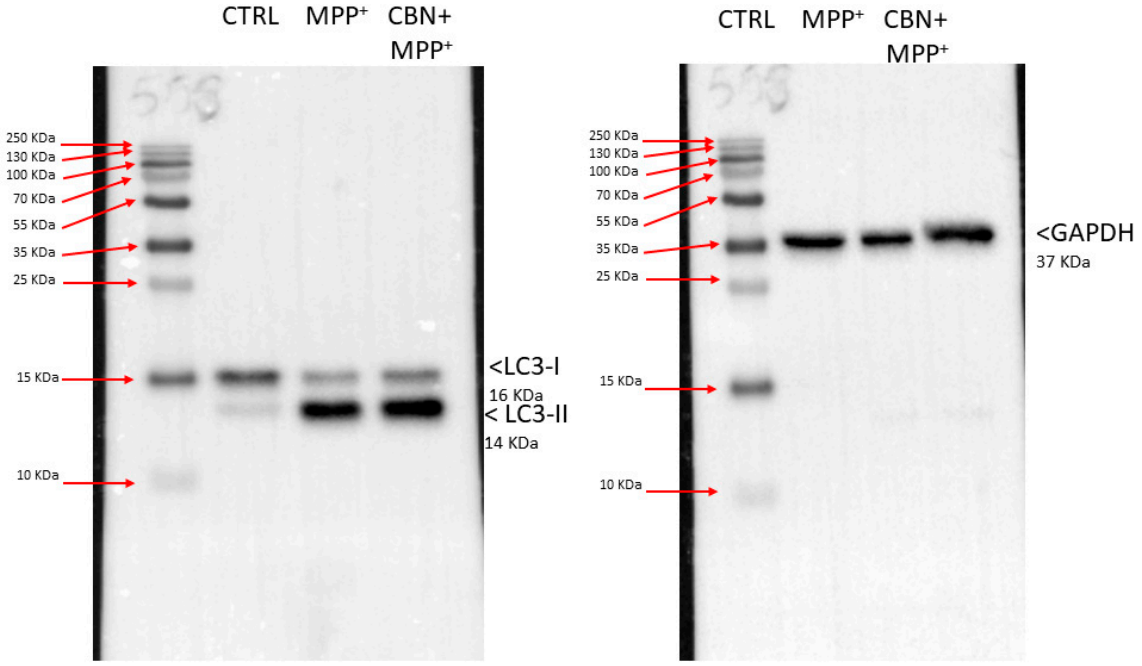

Figure S2: Uncropped blot for LC3 and GAPDH.

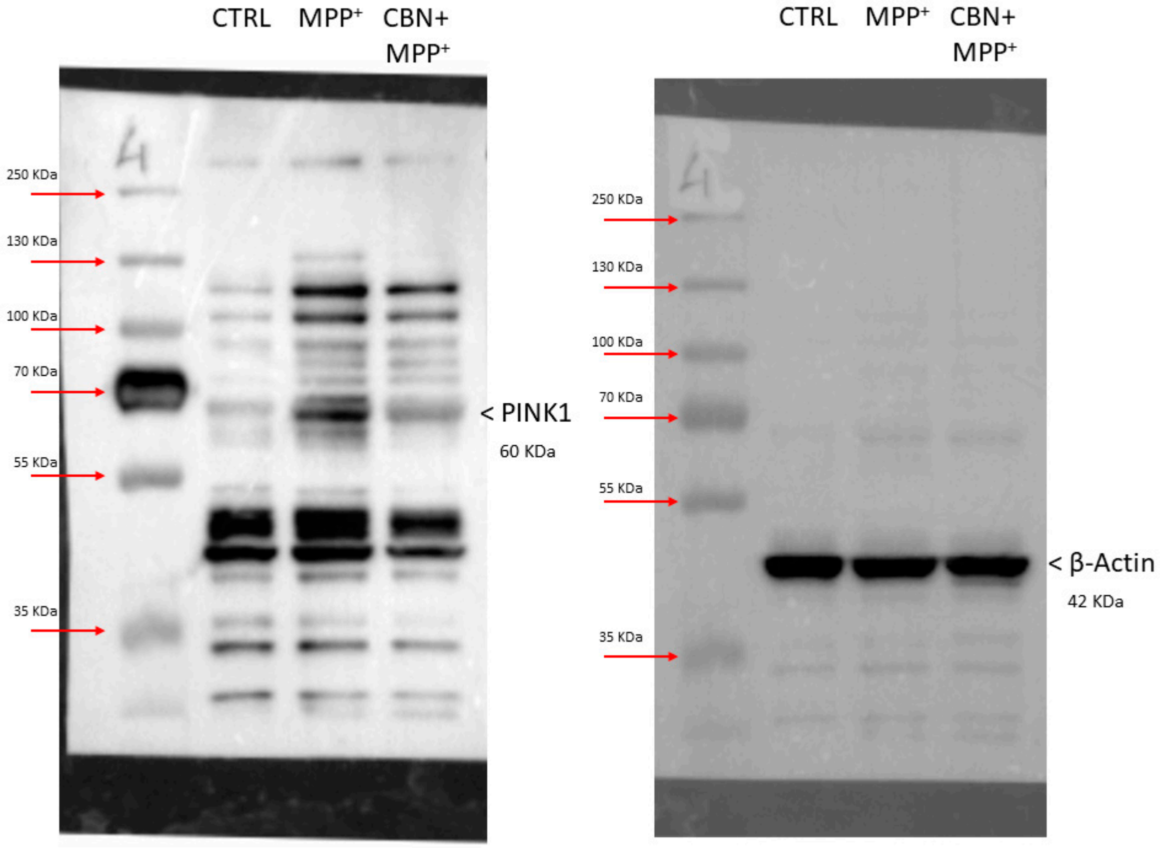

Figure S3: Uncropped blot for PINK1.

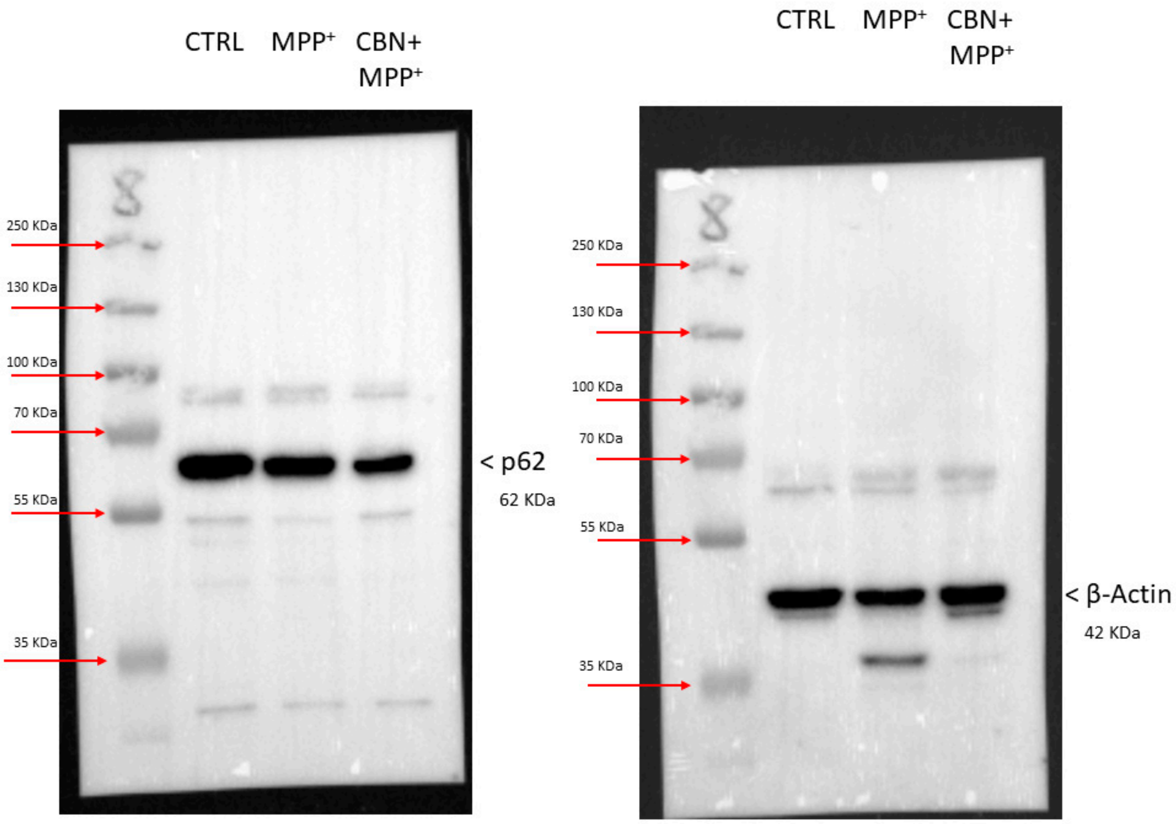

Figure S4: Uncropped blot for p62.
